# Eradication of Benign Skin Lesions of the Face by Voltaic Arc Dermabrasion (Atmospheric Plasma): Postoperative Pain Assessment by Thermal Infrared Imaging

**DOI:** 10.1007/s00266-020-01891-z

**Published:** 2020-08-06

**Authors:** Antonio Scarano, Francesco Carinci, Valentina Candotto, Felice Lorusso

**Affiliations:** 1 Department of Medical, Oral and Biotechnological Sciences and CAST, University of Chieti-Pescara, Via Dei Vestini 31, 66100 Chieti, Italy; 2 University of Ferrara, Ferrara, Italy; 3 Department of Biomedical, Surgical and Dental Sciences University of Milan, 20122 Milan, Italy

**Keywords:** Plasma, Atmospheric plasma, Rhytides, Dermabrasion, Electrosurgery, Skin lesions, Voltaic arc dermabrasion

## Abstract

**Objectives:**

The face aging processes are associated with physiologic and biochemical alteration that produces wrinkles, skin pigmentation and benign growths. The aim of this study was to evaluate the clinical efficacy of voltaic arc dermabrasion with plasma to remove benign facial skin lesions.

**Study Design:**

Voltaic arc dermabrasion plasma technique was used to remove the facial benign skin lesions. The study involved 45 patients (26 females;19 males) treated for benign facial skin lesions with voltaic arc dermabrasion also called plasma exeresis technique. The subjects age ranged between 43 and 65 years. The clinical observations and comparison of pretreatment and post-treatment photographs of the treated regions were performed by a joint examiner at each follow-up visit.

**Results:**

During plasma irradiation, the average temperature of the skin was 290.3 ± 21.7 °C, while immediately after it was 90.6 ± 21.8 °C. Overall clinical improvement was 100% in six lesions with complete resolution of all lesions. Three patients observed a transient post-inflammatory pigmentation with a peak at 1 month after VAD treatment, gradually fading spontaneously over 2 to 3 months.

**Conclusions:**

The voltaic arc dermabrasion technique (atmospheric plasma) should be considered for lesions, especially relatively superficial ones, and small lesions that are located on the face.

**Level of Evidence IV:**

This journal requires that authors assign a level of evidence to each article. For a full description of these Evidence-Based Medicine ratings, please refer to the Table of Contents or the online Instructions to Authors www.springer.com/00266.

## Introduction

The aging process of the face is a gradual atrophic progression of soft and hard tissues [1] and takes place gradually, over 3 to 4 decades, with little clinical evidence. It is eventually recognized by the emergence of furrows and wrinkles together with a loss of tonicity. Other cutaneous signs also appear with aging and are, in part, the result of photoaging and onset benign lesions. It is common for patients to go to an aesthetics surgery practice for the esthetic removal of nevi and various other benign lesions, sebaceous hyperplasia, syringoma, dermatosis papulosa nigra (DPN), skin tags (acrochordons) and verrucae including keratosis. If located on the face, they cause problems for the patient’s self-esteem and can become important enough to affect the quality of life in psychological and in sociocultural terms. There are several basic causes of benign skin lesions on the face: aging of the skin, heredity, post-traumatic effect, hormonal failure, pregnancy, diabetes mellitus, improper nutrition and overweight, stomach problems, severe sweating [2]. In most cases, they are represented by fibrohistiocytic masses with an unknown etiology [3]. The facial nevus (FN) is a nonpigmented or pigmented benign tumor of the skin that contains nevus cells and is present on virtually all adults and a vast majority of moles. Different techniques have been used for removing fibroids and other benign skin lesions: electrocoagulation, laser removal, removal using liquid nitrogen, surgical excision, freezing and radiowave coagulation [4, 5]. Dermabrasion is applied to superficial lesions on the face, and it can be considered as safe to the level of the superficial or mid-reticular dermis [6]. This technique has been proposed with success for the treatment of wrinkles [7] and skin lesions [8]. The benefit of the dermabrasion plasma technique is that it requires a relatively low cost for the equipment, but presents disadvantages related to a potential exposure of the surgeon to blood-borne pathogens aerosolized by the dermabrading procedure [9]. Voltaic arc dermabrasion (VAD) also called atmospheric plasma is a new method used with success for skin resurfacing [10], and it can yield excellent results when a well-trained surgeon performs the procedure to remove skin lesions [11]. The present study was based on the hypothesis that plasma exeresis removes benign facial skin lesions without damaging the surrounding tissues with a restitutio ad integrum of the skin.

The aim of this study is to evaluate the clinical efficacy and safety of voltaic arc dermabrasion with plasma to remove benign facial skin lesions. This involved a clinical evaluation of erythema and pain during procedure by infrared thermal imaging (i.e., thermography) and visual analogue scale. The null hypothesis stated that there is no damage to the surrounding tissues nor permanent inflammatory hypo/hyperpigmentation of the skin.

## Materials and Methods

**Fig. 1:**
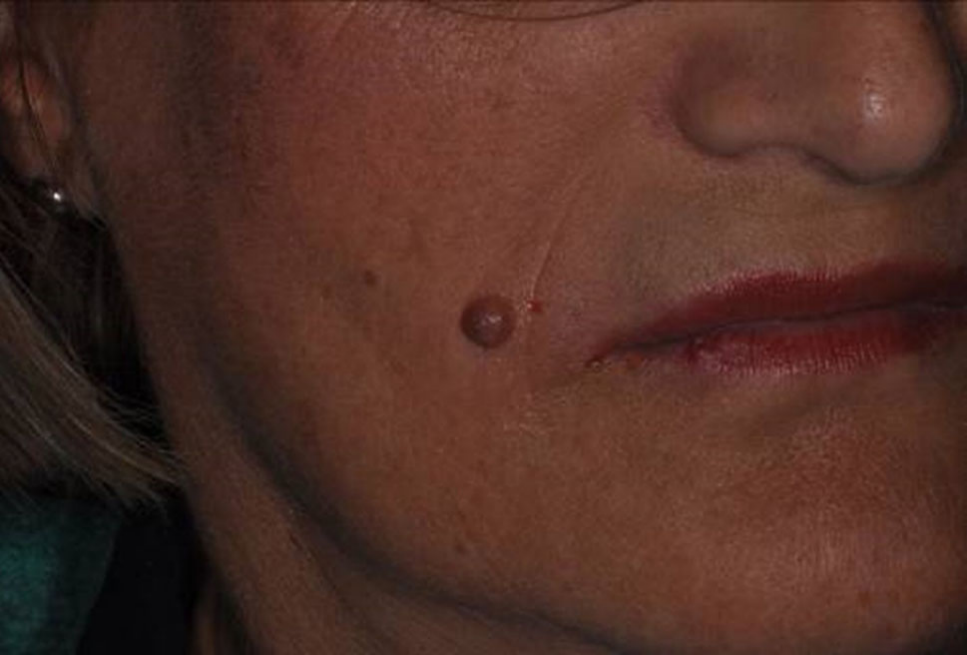
Before treatment of dermal nevus of cutaneous portion of the right perioral area

**Fig. 2:**
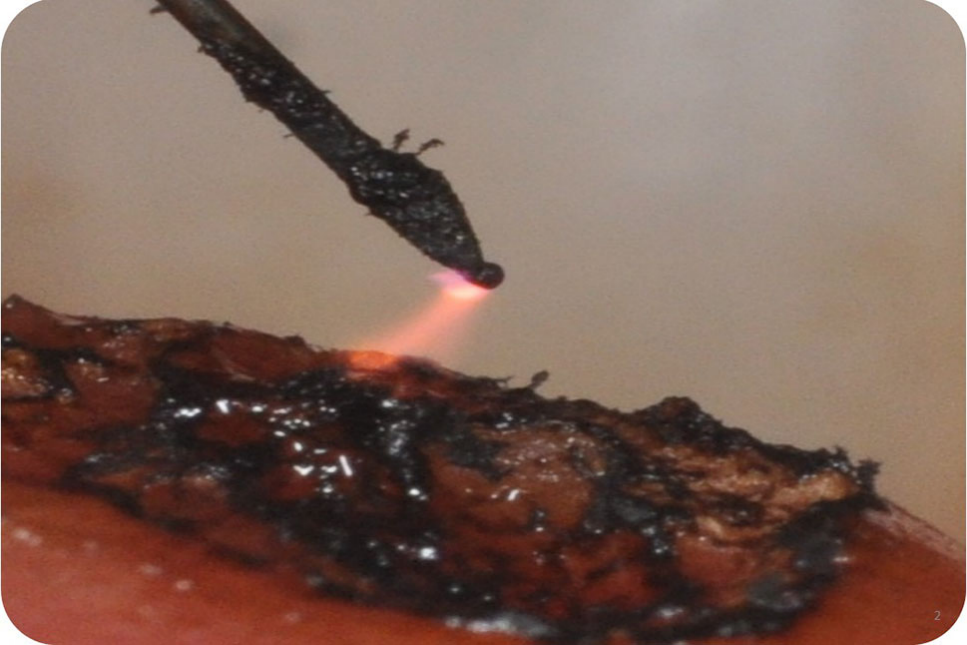
During plasma exeresis of dermal nevus with atmospheric plasma. The pin does not work if held in direct contact with the tissue to be treated, since it requires a small gap to be left for the generation of the plasma forming electric arc. VAD produces a carbonaceous residue layer formed after plasma exeresis

**Fig. 3:**
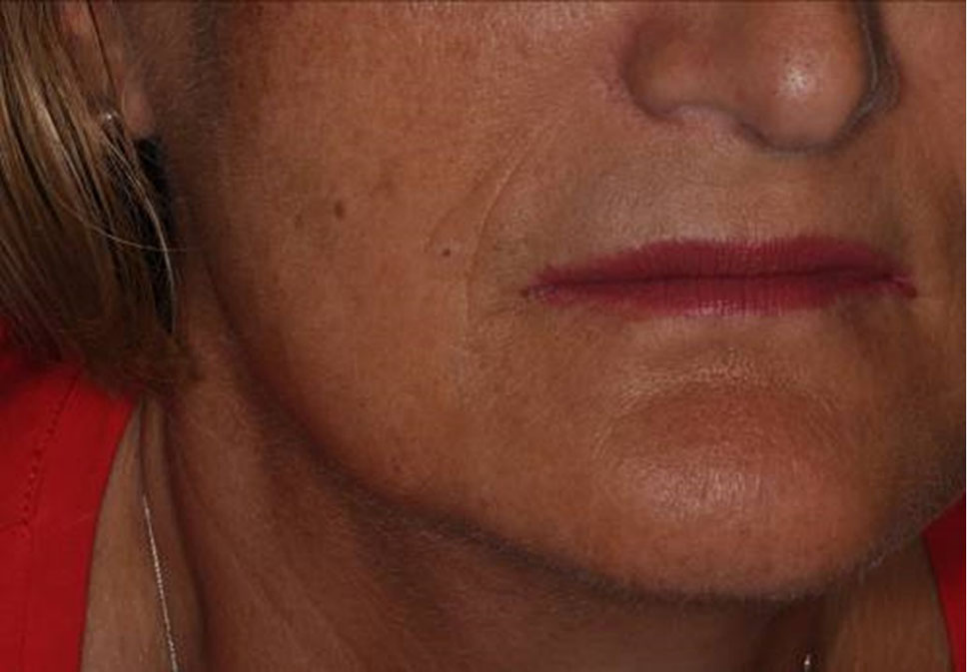
Appearance of a typical patient 30 days after undergoing dermabrasion with voltaic arc technique

**Fig. 4:**
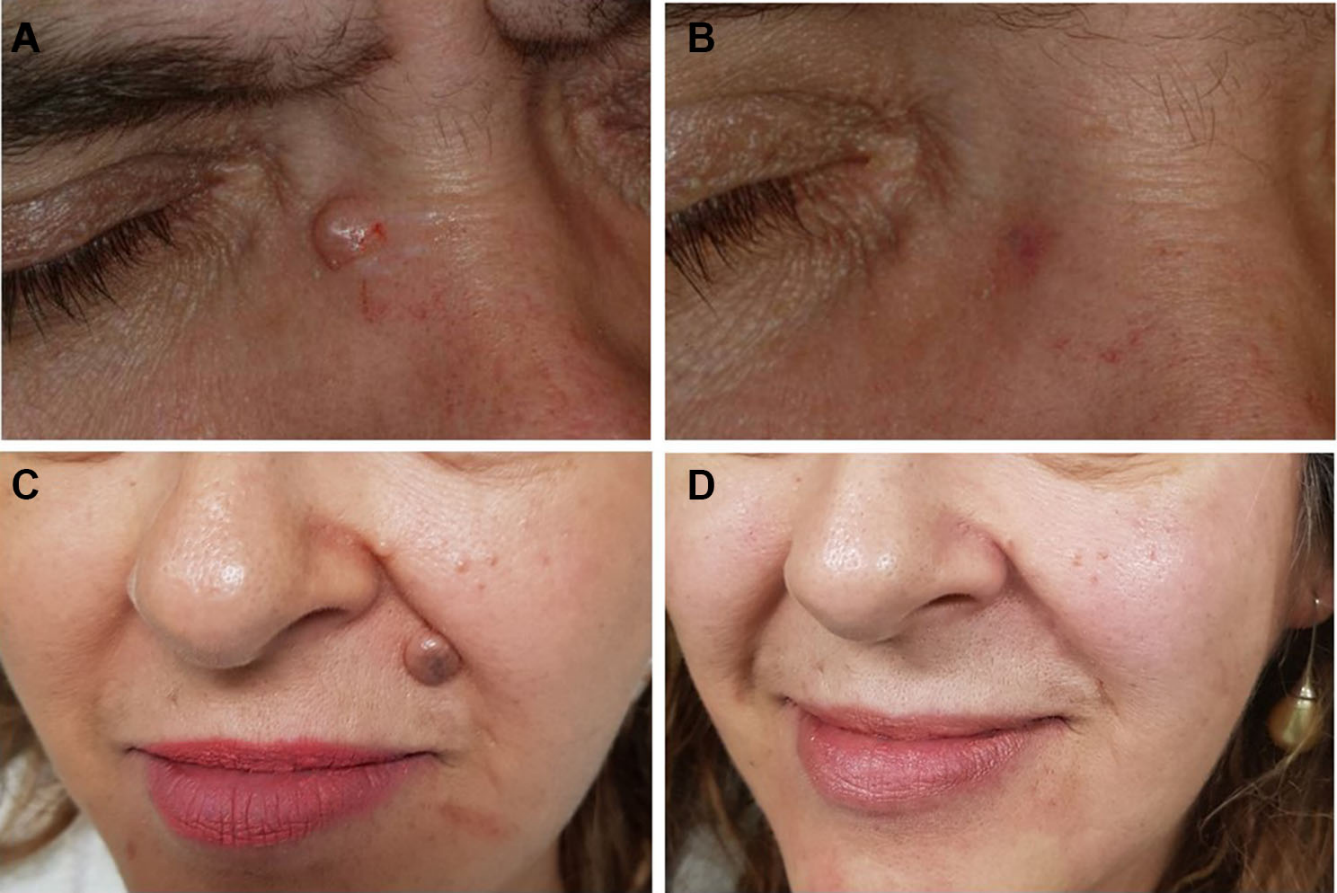
**a** Perinasal dermal nevus after local infiltration anesthesia. **b** Appearance of a typical patient 7 days after undergoing dermabrasion with voltaic arc technique. **c** Perioral dermal nevus before treatment**. d** Appearance of a typical patient 30 days after undergoing dermabrasion with atmospheric plasma technique

The authors treated 45 patients (26 females, 19 males) with VAD plasma procedure to eradicate benign facial skin lesions (Figs. 1–4). The age of the subjects ranged between 43 and 65 years.

Three patients had Fitzpatrick skin type IV, 23 patients had type III, and 19 patients had type II. The pigmentation (scale of 0–3), size [palpation scale of 0 (not palpable) to 3 (easily palpable)] and diameter (mm) were evaluated before treatment.

Several features may indicate that a skin lesion may be something more than a mole, such as a potential skin cancer. Dermoscopy is a noninvasive, in vivo, imagistic technique for intermediate indication in diagnosis. It was used for clinical diagnosis for detection of malignant skin lesions and identified the high-risk malignant lesions melanoma or basal carcinoma [12]. All suspect skin lesions were excluded from the study. Eighteen patients with clinical and dermoscopic diagnosis of dermatofibroma, 12 patients with epidermal and dermal nevi, 6 patients with neurofibroma, 4 patients with angiofibroma, 3 patients with of sebaceous hyperplasia, 1 patient with actinic keratosis and 1 patient with lipoma were treated with atmospheric Plasma. All risks associated with the technique, possible complications such as bruising, swelling, benefits and alternatives to the procedure were discussed with the subjects, who signed the informed consent form. Antibiotics were not routinely prescribed to patients before the intervention nor postoperatively. Voltaic arc dermabrasion (VAD, Europe Medical s.r.l. Montesilvano (PE), Italy) was used to remove benign facial skin lesions. The device consists of a handheld atmospheric pressure plasma jet, with electrode discharge in atmospheric gas. The electrode was connected to a commercial 50 kHz high voltage alternating current power supply (3 kV, 2 mA) with 2 W. The facial region was injected with Articaine® (Curaden Healthcare S.p.A., Saronno, Italy) associated with epinephrine 1:100.000 as local infiltration anesthesia. The lesions requiring differential diagnosis were shave biopsied just before ablation, by using a scalpel to remove a representative portion of the lesion.

To remove facial benign skin lesions, different steps are required by VAD plasma technique with a crosshatch treatment pattern (Fig. 2). The first phase involves vertical strokes, and the second phase requires horizontal strokes. The technique is non-overlapping and vaporizes by voltaic arc, followed by gentle yet thorough wiping of the desiccated debris with saline-soaked sponges. At each passage, there is a smooth passing through the tissue with plasma (sparking) (Fig. 2). Then, the lesion is firmly wiped with wet gauze to remove the charred tissue. The VAD technique was used for skin lesions that rise above the skin or are in the upper layer of skin. This technique removes the outermost layers of skin lesion layer by layer until the lesion reaches the same level as healthy skin. The lesion’s surface then reveals a pink hue, representing partially denatured papillary dermis. Furthermore, the injection of anesthesia produces swelling and firmness (tumescence) of the skin lesion and produces the separation of tissue planes allowing the surgeon to have an improved plane of ablation. After removal, the patient was instructed to refrain from any manipulation of the wound site and the patient could go immediately back to work. A hypoallergenic fluid foundation was applied after the procedure to protect the area and to cover the carbonaceous residue layer of the treatment (Fig. 2). The results were evaluated at 1, 2 and 3 months after the procedure.

The clinical observation and comparison of pretreatment and post-treatment photographs of the treated regions were performed by a joint examiner at each visit, in order to evaluate the results during the follow-up.

Pain intensity was classified into 4 different categories. Postoperative pain was scored by means of a 100-mm VAS from 0 (no pain) to 100 (worst pain imaginable) at 1-, 4- and 6-day intervals. Erythema was classified into four categories: number 1 stands for the absence of erythema, patients with a perilesion erythema extending for 1 mm scored 2, patients with a perilesion erythema extending for 2 mm scored 3, and intense erythema exhibited by perilesion and erythema extending beyond 3 mm in the treated zone scored 4 [13]. At each visit, standardized photographs were taken, and patients rate their procedure satisfaction following the validated Global Aesthetic Improvement Scale (GAIS):

• Grade 3: Excellent (patient completely satisfied with the result).

• Grade 2: Very good (patient very satisfied with the result).

• Grade 1: Satisfactory (although the patient sees slight improvement, additional correction is required).

• Grade 0: Indifferent (patient sees no changes).

• Grade 1: Unsatisfied (patient’s condition is worse than before the procedure).

Follow-up visits were scheduled at 1, 4 and 6 days to check the state of tissues. The recall program included assessment of VAS, erythema and perilesion temperature. Patients followed short-term assessments at 1, 2 and 4 days and 1, 2 and 3 months, 1 year after the final treatment session.

## Temperature Measurements

Thermal surveys were rated in a climate-controlled room (temperature: 22–24 °C, relative humidity: 50 ± 5%, with no direct ventilation into the mouth of patients). Perilesion temperature of the treated side was obtained by a 14-bit digital infrared camera (FLIR SC660 QWIP, FLIR Systems, Danderyd, Sweden). The parameters of acquisition applied to the measurement were 320 × 240 pixels focal plane array; 8–9 µm spectral range; 0.02 K noise equivalent temperature differences (NETDs); 50-Hz sampling rate; optics: germanium lens; f 20; and f/1.5. The camera distance was set at 0.50 m away from the mouth for maximum spatial resolution. Images were acquired at a rate of 25 10 images per second and subsequently re-aligned using an edge-detection-based method, implemented with in-house software. A video was performed, and photographs were extrapolated via dedicated software. Temperature changes in the perilesion were elaborated on the realigned thermal images. Thermographic data analysis was performed using FLIR QuickReport v.1.2 (FLIR Systems Inc., North Billerica, MA, USA), which includes a tool to obtain maximum, minimum and average temperature of the perilesion area.

### Statistical Evaluation

Data and statistical analysis were elaborated using Excel software (Microsoft Office, Redmond, WA, USA), Origin (OriginLab, Northampton, MA, USA) and SPSS software (IBM, Armonk, NY, USA). The VAS differences, erythema and perilesion temperature before and after treatment were evaluated by descriptive statistical methods and repeated-measures ANOVA statistical analysis followed by Tukey’s post hoc test for significance was performed. A value of *p* ≤ 0.05 was considered statistically significant.

## Results

Overall clinical improvement was 100% in six lesions with complete resolution of all lesions (Figs.3,4). Usually, the removal of the lesions involved an average time of 120 ± 30 s. Immediately after skin lesion removal, the tissue appeared as a pale, erythematous, dull surface and it was coated with carbonaceous residue formed after the skin ablation (Fig. 2). Bleeding was not seen unless excessive abrading occurred with the saline-moistened gauze. At 4 days, the wound area showed a secondary granulation with slight swelling and reddened wound edges covering the area.

After 1 month’s post-surgery, the esthetic state was excellent and only marginally raised erythema was noticeable. Three patients observed a transient post-inflammatory pigmentation with a peak at 1 month after VAD treatment, still spontaneously fading gradually over 2–3 months.

No erythema, ecchymosis, itching, outbreaks of herpes, infectious processes or scarring were observed. Swelling was not recorded after skin treatment. Based on the VAS scores, sometimes a mild pain was recorded only after skin lesion ablation and none reported a moderate or severe pain after VAD treatment. All patients presented well with no occurrence of symptoms indicating possible perilesional inflammation.

Based on the VAS scores, very mild discomfort during plasma irradiation was reported in all patients with an average pain score of 3.21 ± 1.72. No pain or discomfort was recorded after plasma irradiation and at 1, 4 and 6 days after the procedure. No statistical difference was recorded before and after plasma irradiation *(p* < 0.5). No outbreaks of herpes, ecchymosis, hypopigmentation, hyperpigmentation, erythema, itching, infectious processes or scarring were observed. Erythema, as a typical sign of heat application to the superficial skin, was present to a minimal extent only with an average 0.4 ± 0.1 immediately after VAD irradiation and was decreased at 1 (0.3 ± 0.2), 4 (0.3 ± 0.1) and 6 (0.2 ± 0.2) days (Fig. 7). Slight erythema was recorded at 6 days (Fig. 4b).

**Fig. 5:**
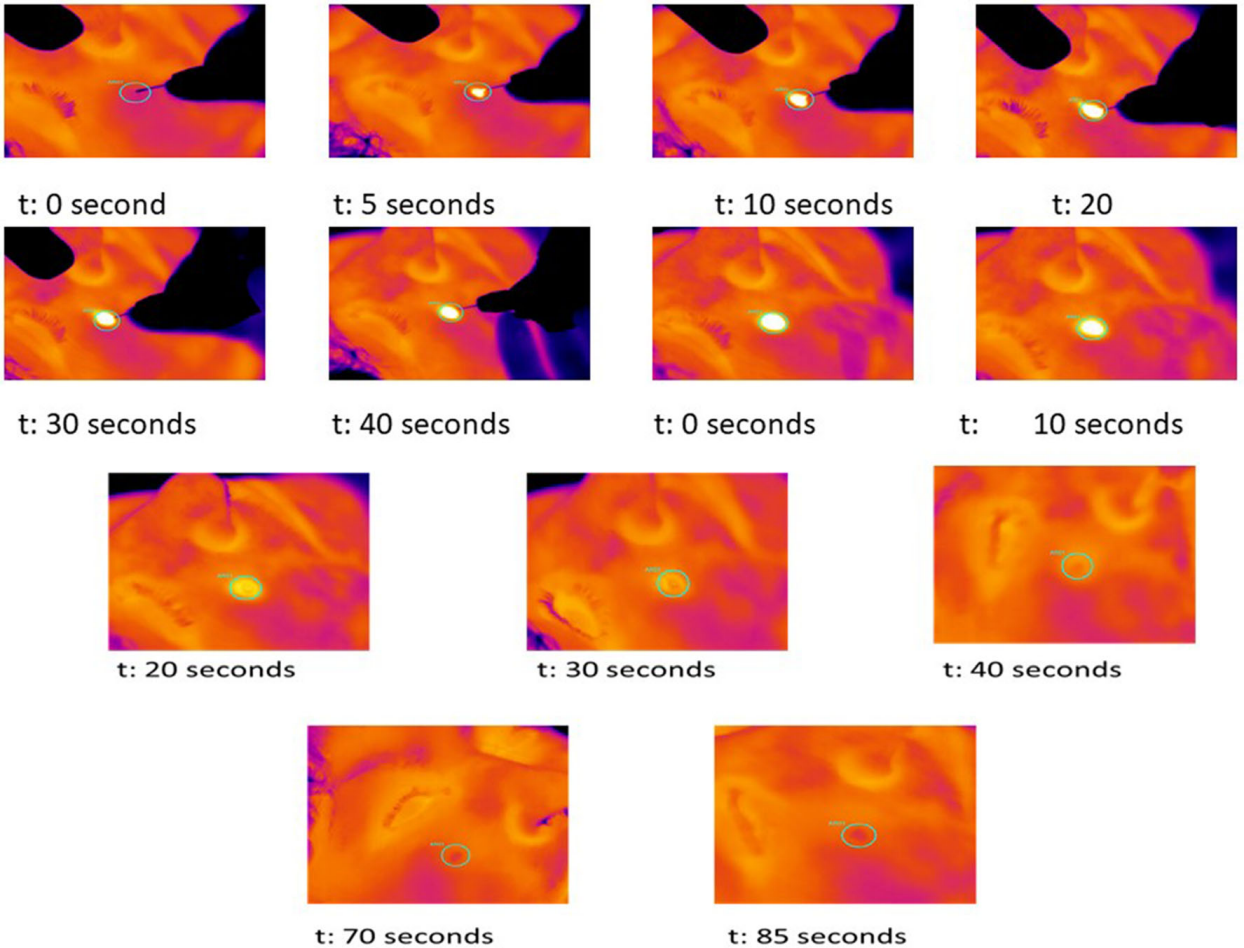
Measuring skin temperature with infrared thermal camera during and after atmospheric plasma exeresis. The temperature returned to normal after 90 s. The dimensions of image are 11.8 × 12.7 × 7.2 mm, while the spatial resolution of interrogation area is 320 × 240 pixels

**Fig. 6:**
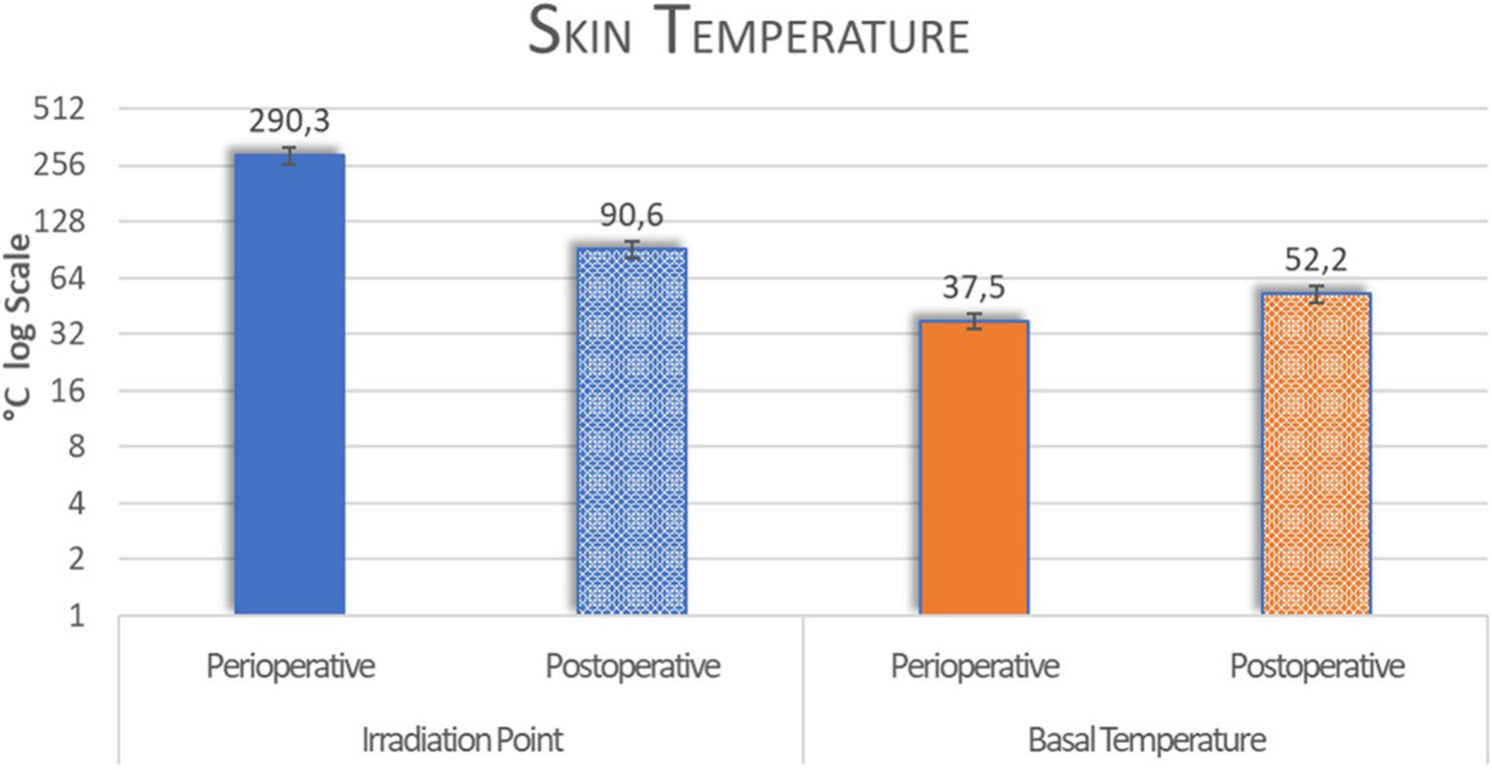
Temperature evaluated on irradiated point and skin basal temperature

**Fig. 7:**
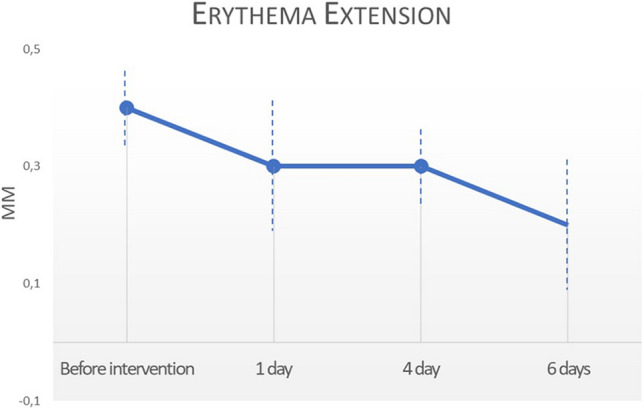
Erythema extension before intervention, at 1, 4 and 6 days

During plasma irradiation, the average temperature of the skin was 290.3 ± 21.7 °C, while immediately after it was 90.6 ± 21.8 °C (Figs. 6,7). The difference in temperature before the procedure (basal measurement 37.5 ± 2.8 °C) 37.5 ± 2.8 °C and immediately after plasma irradiation was 52.2 ± 4.5 °C (Figs. 6,7 and Tables 1, 2). The temperature lowered for the most part in 10 s and completely normal after 40 ± 0.25 s (Figs. 5,6).

**Table 1:** VAS score and temperature measurement at different times by mean and standard deviations

Vas Score	Perioperative	Before intervention	1, 4, 6 days
Mean	3.21	0.0	0.0
Standard deviation	± 1.72	–	–
*p* value	*p* < 0.01		–
CI 95,00%	(− 3.743 to − 2.677)		–

Temperature	Perioperative Irradiation Point	Before Irradiation Point	Perioperative Basal measurement	Postoperative Basal measurement
Mean	290.3 °C	90.6 ± 21.8 °C	37.5 °C	52.2 °C
Standard deviation	± 21.7	± 21.8	± 2.8	± 4.5
*p* value	*p* < 0.01		*p* < 0.01	
CI 95,00%	(− 208.8 to − 190.6)		(7.075 to 10.32)	

The VAS score mean was 0.0 at 1, 4 and 6 days, and the symptoms were absent for all subjects enrolled at the indicated timepoints.

**Table 2:** Erythema extension at different times by mean and standard deviations

Erythema	After intervention (A)	1 day (B)	4 day (C)	6 days (D)
Mean	0.4 mm	0.3 mm	0.3 mm	0.2 mm
Standard deviation	± 0.1	± 0.2	± 0.1	± 0.2

	Mean Diff,	CI 95,00%	*p *value
(A-B)	0.1000	(0.03054 to 0.1695)	*p* > 0.05
(A-C)	0.1000	(0.06111 to 0.1389)	*p* > 0.05
(A-D)**	0.2000	(0.09034 to 0.3097)	*p* < 0.01
(B-C)	0.000	(− 0.06789 to 0.06789)	*p* > 0.05
(B-D)	0.1000	(− 0.01385 to 0.2138)	*p* > 0.05
(C-D)	0.1000	(− 0.007338 to 0.2073)	*p* > 0.05

^**^p < 0.01

Among 45 patients treated with the VAD plasma technique, none referred to be unsatisfied or indifferent during the course of the treatment and a Grade 3 of GAIS was recorded. In fact, the number of patients that referred an “excellent” outcome was thirty at 1 month and the other fifteen at 2 months. A single sitting of plasma irradiation was needed for total eradication of the skin lesions. No difference was observed among all 45 treated cases, although the big dimension lesions seem to heal more slowly. No difference between wound size and erythema was observed.

## Discussion

The results of this study show that a single sitting of plasma irradiation was sufficient for total eradication of the skin lesions. Our results demonstrate that sequential temperature of skin is dissipated rapidly; this shows that deep tissues are marginally heated therefore decreasing the risk of hyperchromia or hypochromic lesions. A very mild discomfort during plasma irradiation with an average pain score of 3.21 ± 1.72 was recorded. This shows that the plasma technique used in this study is not very aggressive; in fact, no erythema, ecchymosis, itching or scarring were observed. No damage to the surrounding tissues nor permanent inflammatory hypo/hyperpigmentation of the skin was recorded. This result shows the efficacy of the plasma technique against skin lesions. The results of the present study support the rejection of the null hypothesis.

Different techniques were used to ablate benign lesions of the neck and head, including common CO_2_ laser, electrosurgery, cryotherapy and surgical excision.

Surgical excision carries the risk of scarring, which makes skin grafting with secondary scar correction necessary. However, the advantage of surgical excision is its histological safety, which allows for a clear histological differentiation thanks to including healthy tissue edges [14].

In a previous study, the VAD plasma technique was used with success to remove xanthelasmas [11] and improving perioral rhytides [15].

Electrosurgery consists in the induction of a high voltage alternative electric current to a biological tissue, thus producing a thermal effect and achieving an incision or coagulating effect [16]. This technique is used for soft tissue surgery, that can ablate leaving a 100–400 µm necrotic tissue layer.

The electrosurgery is based on the ability of the current to pass through the tissue and attain the temperature for the desired effect on the target [17]. For any electrical circuit to exist, there must be an active electrode and dispersive electrode pole to create the conditions for movement of the electric current. The procedure consists in one electrode mounted on the device, where the entire patient is interposed between this “active electrode” and the large dispersive electrode located relatively distant from the target tissue, typically on the thigh or back. The active electrode in contact with tissue generates heat, resulting in thermal tissue damage, due to resistance to the passage of the flow of the current. But the electric current passes preferably through low-resistance tissues. Vascular tissue and hydrated skin are good conductors, so they are easy to treat with electrosurgery, but a dry skin and connective tissue are unlikely to be crossed by an electric current; if the target is a poor conductive tissue, it will be difficult for the current to pass.

To overcome this problem, VAD plasma technique was proposed. In physics, the term “plasma” is typically used to name the fourth state of matter apart from the solid, liquid and gas states and is a highly active ionized gas. The voltaic arc acts without getting into tip-tissue contact, creating a gentle coagulation. There is no electric passage zone; for this reason, plasma dermabrasion is not influenced by the tissue’s resistance to the current. Voltaic arc is a process whereby the tissue is superficially coagulated by repeated electrosurgical voltage arcs that continue to elevate the temperature by resistive heating without dispersive electrodes. In this case, there is not a passage of current in the tissue and the electrode is near to but not in contact with the tissue. The gas between tissue and electrode is nonconductive; however, when a high voltage electric field is used, the gas will be ionized and becomes plasma and conducts the electrical current as a spark. This causes destruction of a superficial layer of tissue near the electrode. The increase in temperature extends to the surface and not in depth. For this reason, no cases of hypopigmentation, hyperpigmentation or pain were observed. In fact, when using a VAD plasma technique, there is not a deep current density passage which could consequently descend to the depth of dermal damage and the possibility of visible scar formation.

During the operation, it is important to be protected by masks to avoid viral particle inhalation [18].

Voltaic arc dermabrasion technique was found to be effective and safe in the treatment of benign lesions on the face. In most cases, the healing process appears rapid, with minimal evidence of pain and erythema that resolved within 20–30 days; any possible untoward effects were relatively few and short-lived.

The benefits of voltaic arc dermabrasion procedure are related to unnecessary postoperative care and a decreased risk of hypopigmentation (i.e., destruction of melanocytes), hyperpigmentation of the treated area [11]. Postoperatively, minimal edema resolves within several hours. In many studies, a high rate of postoperative hypo- and hyperpigmentation was reported, while a much lower incidence was found in this study [19–22]. Other advantages of this technology are that it has a much lower cost than a laser and in its simplicity of use with a very rapid learning curve. Voltaic arc dermabrasion induces a rapid heating of the skin, with limited tissue ablation and minimal collateral thermal damage [10]. A rabbit model study reported the capacity of controlling the tissue removal depth induced by voltaic arc dermabrasion or ablation device and the potential difficulties related to create a precise skin removal using a radiosurgical unit [10]. VAD was used with success to remove or improve facial rhytides [23]. Recently, it has also been used for eyelid blepharoplasty [24], for treating acne volgare [25] and epidermoid cysts [26]. After skin ablation, an epidermal regeneration occurs within 7 days postoperatively that presents aspects of neocollagenesis, visible in histology at 30 days [27]. Since skin-specific quality of life significantly improved after VAD treatment, this therapy can be recommended for patients to eradicate benign skin lesions and to improve their appearance. The VAD (atmospheric plasma) is a novel concept, and for a busy aesthetic surgeon, it is a possible easy and quick solution that eliminates benign skin lesions although it does not replace laser technology. In the present study, we have not compared the use of CO_2_ laser vs VAD, but it seems that the use of the atmospheric plasma offers an excellent opportunity for substantial removal of skin lesions with a rapid healing period. Moreover, there is no evidence that VAD technique is a quicker device than CO_2_ laser. In conclusion, VAD or atmospheric plasma technique is a new method used with success for eliminating benign skin lesions.
